# Antimicrobial Stewardship in the Pediatric Emergency Department: An Observational Pre-Post Study

**DOI:** 10.3390/children12010046

**Published:** 2024-12-30

**Authors:** Erika Silvestro, Ilaria Mussinatto, Antonia Versace, Marco Denina, Giulia Pruccoli, Raffaella Marino, Giulia Mazzetti, Lorenzo Scaglione, Federico Vigna, Alessandra Macciotta, Silvia Garazzino, Claudia Bondone

**Affiliations:** 1 Infectious Diseases Unit, Department of Pediatrics, Regina Margherita Children’s Hospital, A.O.U. Città della Salute e della Scienza di Torino, 10126 Turin, TO, Italy; esilvestro@cittadellasalute.to.it (E.S.); gpruccoli@cittadellasalute.to.it (G.P.); rmarino@cittadellasalute.to.it (R.M.); giulia.mazzetti@unito.it (G.M.); lorenzo.scaglione@unito.it (L.S.); federico.vigna@unito.it (F.V.); sgarazzino@cittadellasalute.to.it (S.G.); 2 Division of Pediatrics and Neonatology, P.O. Chivasso, ASL TO4, Corso G. Ferraris 3, 10034 Chivasso, TO, Italy; imussinatto@aslto4.piemonte.it; 3 Department of Pediatric Emergency, Regina Margherita Children’s Hospital, A.O.U. Città della Salute e della Scienza di Torino, 10126 Turin, TO, Italy; aversace@cittadellasalute.to.it (A.V.); cbondone@cittadellasalute.to.it (C.B.); 4 Department of Clinical and Biological Sciences, University of Turin, 10126 Turin, TO, Italy; alessandra.macciotta@unito.it

**Keywords:** antimicrobial stewardship, pediatric emergency department, pediatric infectious diseases

## Abstract

**Background/Objectives**: To face antimicrobial resistance, antimicrobial stewardship programs (ASPs) have been implemented in the pediatric age, but the area of urgency remains understudied. We aimed to assess the impact of an educational program on optimizing antibiotic appropriateness in a pediatric ED. **Methods**: We conducted a pre-post observational study with an audit, intervention, and feedback given to prescribers. We recorded all systemic antibiotic prescriptions for children attending our pediatric ED from January to March and from July to September 2020. The study’s team assigned a score to each prescription, regarding the appropriate molecule, dose, and duration of therapy, according to the diagnosis. From April to June 2020, we held weekly meetings focusing on different pediatric infectious diseases, with interaction between one to three ED physicians and the infectious disease (ID) specialist of the study’s team. We then distributed synthetic digital guidelines adapted to our reality to all prescribers. **Results**: Optimal antibiotic prescriptions increased after the intervention, with statistical significance (*p* < 0.001) in four main aspects (overall adequacy: 13% PRE vs. 43% POST; need of antibiotics: 53% vs. 68%; adequacy of the spectrum: 55% vs. 63%; adequacy of the chosen molecule: 54% vs. 62%). We observed an improvement in all the main infectious diseases and concerns all the ED physicians. The prescription of first-choice drugs increased in specific and common illnesses such as otitis and pharyngotonsillitis. **Conclusions**: An antimicrobial stewardship program is a relevant method for improving the appropriateness of antimicrobial use also in the complex setting of a pediatric ED.

## 1. Introduction

The use of antibiotics in hospital and outpatient settings has increased worldwide, but the antibiotic therapies prescribed for common infections are not always considered necessary [1,2].

Overuse of antibiotics results in additional costs and avoidable adverse effects. The most worrisome consequence is the remarkable and worldwide increase in multidrug-resistant (MDR) pathogens. Resistant isolates are no longer confined to healthcare settings but have emerged in the community [3].

To improve the use of antibiotics, strategies known as antimicrobial stewardship programs (ASPs) have been developed in recent years: they consist of a series of coordinated and multidisciplinary interventions aimed at promoting the most appropriate use of antibiotics in a specific clinical setting, focusing on the optimal choice of drug, dose, duration of therapy and route of administration. This approach is widely used in many clinical settings and is a fundamental tool in the prevention of antimicrobial resistance [4,5].

For both adults and children, the emergency department (ED) presents unique complexities due to the scarcity of microbiologic results, the need to make quick decisions, high patient and physician turnover rates, and concerns about patient satisfaction [6]. In addition, in the emergency department, most patients are discharged, so antibiotic therapy management resembles that in the local area, with little or no opportunity for de-escalation at 48–72 h as would be recommended [7]. Despite these difficulties, ED physicians play a pivotal role in selecting the most appropriate empiric therapy, with a downstream effect: therapeutic regimens initiated in the ED are often continued with reluctance to de-escalate by another provider [8].

Some studies show that it is possible to reduce antibiotic prescriptions in the ED, both in adults and children [9,10]. However, despite the widespread use of ASPs projects, the area of urgency, particularly pediatric urgency, remains understudied [11].

Our study aims to describe antibiotic prescribing in our pediatric emergency department and to evaluate the impact of an educational intervention on optimizing antibiotic appropriateness. The development of ASPs tailored to the pediatric emergency setting is essential to improve the approach to every child presenting to the healthcare system with a suspected bacterial infection. Emergency physicians play a key role in the implementation of ASP initiatives.

## 2. Materials and Methods

We conducted a pre-post observational study consisting of an audit followed by an intervention based on interactions between prescribers and ID specialists and feedback provided to prescribers. A clinical pharmacist trained in infectious diseases was also involved: his role consisted of providing support to the stewardship team on the most appropriate antibiotic formulations for pediatric administration and the appropriateness of the drug dose per kg of weight of the child, and guidance on the molecules actually present at our hospital and therefore prescribable to patients.

We conducted the study in the pediatric ED of the Regina Margherita Children’s Hospital, a tertiary care academic pediatric hospital in Turin, Italy. Approximately 40,000 children aged 0–18 years visit our pediatric ED annually for acute surgical or medical conditions.

We conducted a prospective study of all children (0–14 years) attending the medical, pediatric ED of our hospital for 3 months (January–March 2020), recording data of all patients who received a systemic antibiotic prescription. Each prescription was then evaluated by an ID specialist and a pediatrician, with the assignment of an appropriateness score, following the most recent international and local guidelines for each pediatric infectious disease, regarding six items: the overall appropriateness of the prescription, the need for antibiotics according to the diagnosis, the appropriateness of the spectrum, the adequacy of the molecule chosen, the prescribed dose, and the duration of therapy. The score consisted of 3 levels of agreement: A, complete agreement; B, moderate agreement; C, disagreement (see Supplementary File).

From April to June 2020, we carried out the intervention phase. We held a weekly meeting focusing on a different pediatric infectious disease, starting from discussing clinical cases treated in the ED. The participants were a small number (1–3) of ED prescribing physicians in the presence of an ID physician. These meetings allowed us the possibility to highlight the main difficulties encountered in the daily management of patients and to improve diagnostic and therapeutic pathways according to local and international guidelines. We discussed pharyngitis, otitis, pulmonary infections, urinary tract infections, soft tissue infections (impetigo, skin abscess, cellulitis), osteomyelitis, meningitis and encephalitis, lymphadenitis, abdominal infections, and other oral infections.

After each meeting, a summary document was prepared with the topics covered, relevant national or international guidelines for proper antibiotic treatment with respect to the disease, and resistance data by pathogen available at our hospital. This document was then circulated to all medical staff in the emergency department. We reported the pathogen, the empirical first- and second-line therapy, the definitive therapy, the most appropriate dose, the timing of parenteral to oral conversion, and the microbiological test available or diagnostic score. The digital distribution of this material has allowed 100% dissemination to prescribers and easy smartphone consultation.

From July to September 2020, we conducted the post-intervention phase, in which we again collected data from all children who received a systemic antibiotic prescription with the same scoring system applied.

We entered anonymous patient data into an electronic spreadsheet database (Excel 2008 [Microsoft, Redmond, WA, USA]), and we performed statistical analysis using the R software (version 3.6.2). We used the *t*-test for continuous data or the χ^2^ test/Fisher’s exact test for categorical data, depending on the number of samples, as appropriate. The alpha significance level was set at *p*-value ≤ 0.05.

## 3. Results

### 3.1. Study Population and Descriptive Data

During the first study period (winter), 4610 children visited the ED for medical reasons, and 1128 (24.4%) of them received a prescription for systemic antibiotics, both for home treatment and for hospital admission. In the second period (summer), a total of 2221 children visited the pediatric ED for medical reasons, and 294 (13.2%) of them received a prescription for systemic antibiotics. Table 1 shows the characteristics of the study population.

In both periods, the preferred route of administration of antibiotic therapy was oral. There is a statistically significant difference in the route of administration chosen between the two periods, with more use of the parenteral route in the second period. Therapeutic management also differed significantly between the two periods, with a higher percentage of hospitalizations in the summer period than in the winter period (17.4% POST vs. 13.1% PRE; *p* < 0.009).

Upper respiratory tract infections were the main cause of antibiotic prescriptions in both the winter and summer.

**Figure 1:**
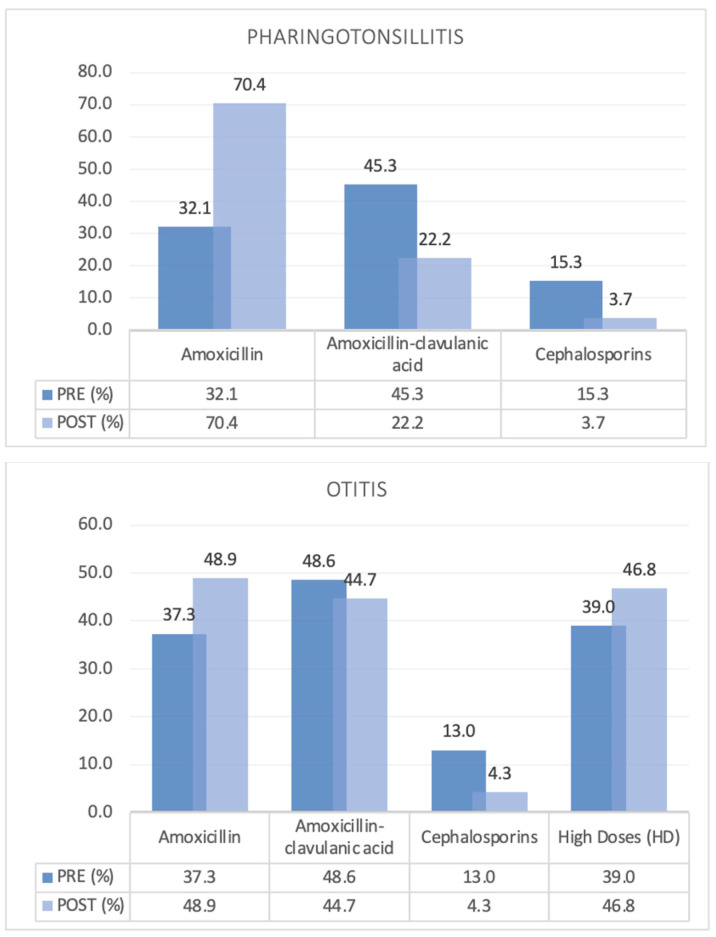
Common antibiotics used to treat pharyngotonsillitis and otitis media, pre–post comparison.

Amoxicillin–clavulanic acid was the most commonly used antibiotic both before and after the intervention. The ratio of amoxicillin/amoxicillin–clavulanic acid was 0.6 PRE vs. 0.4 POST. This difference also reflects a different and statistically significant use of broad-spectrum antibiotic molecules, which increased in the POST period (Table 2). However, when analyzing the data for specific and common infectious pathologies, we observed a reversal of the amoxicillin/amoxicillin–clavulanic acid ratio in pharyngotonsillitis (0.7 PRE vs. 3.1 POST) and otitis (0.76 PRE vs. 1.09 POST) (Figure 1).

### 3.2. ASP Activity

**Figure 2:**
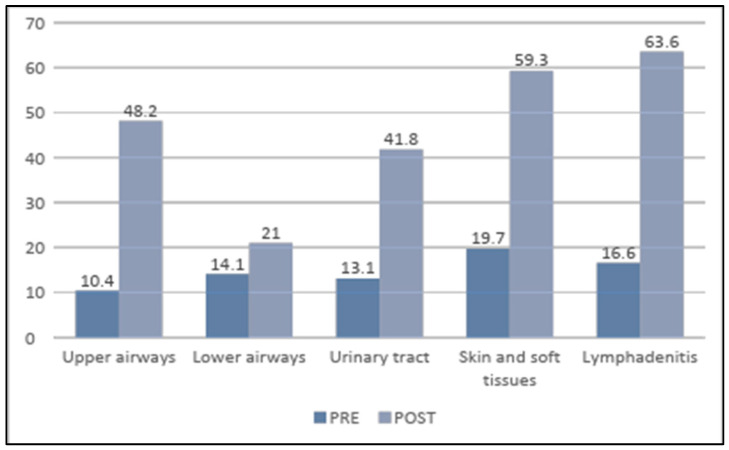
Percentage of grade “A” (complete agreement with the prescription by the study team) for the item “overall prescription appropriateness” for the site of infection, pre-post comparison.

All six prescription items showed a difference in the PRE compared to the POST period, with an improvement in five of them (Table 3). This difference was significant in four main aspects (overall appropriateness, need for antibiotics, appropriateness of the spectrum, and of the molecule chosen). This improvement remains evident even when the cases are divided by the site of infection (Figure 2) and for each of the 14 ED prescribers in the ED, including the minority of those (10%) who did not participate directly in the meetings with the ID specialist and only received the digital material.

The prescribed dose was lower than optimal in the first period (69% of cases), while it was higher than necessary in the control period (64.3% of cases). The duration of therapy was shorter in most cases in both the PRE and in the POST periods (Table 3).

In both periods, prescribers preferred molecules with a stronger effect than necessary (Table 2).

## 4. Discussion

Antibiotics are very commonly used in the pediatric population due to the high prevalence of infectious diseases in this age group: despite wide variation between pediatric hospitals (38–72%), Gerber et al. found that 60% of children received at least one antibiotic during their hospital stay [12]. Between 20% and 50% of pediatric antibiotic prescriptions are potentially unnecessary or inappropriate [13,14,15,16], and recent studies have shown that resistance rates have increased in this age group over the last decade [17].

Italy has one of the highest rates of antimicrobial resistance in Europe and is also the European country with the highest consumption of antibiotics. The prevalence of MRSA and vancomycin-resistant E. faecium (VRE-faecium) is higher than the European average, as is resistance to carbapenems in Pseudomonas aeruginosa, Klebsiella pneumoniae, and Acinetobacter [18].

Antibiotics are the most commonly prescribed drugs in the Italian pediatric population [19,20]. The prevalence of use of systemic antibiotics in 2022 was 33.7% of the population aged 0–13 years. Penicillin combinations are the most commonly used class of antibiotics (17.3%), followed by macrolides (9.8%). In 2021, there was a lower use of systemic antibiotics in the pediatric population than in 2019 (23.7% versus 40.9%), probably related to the COVID-19 pandemic, while the following years saw a new increase. Based on the WHO AWaRe classification, more than 40% of the prescriptions in this population were not first- or second-choice antibiotics in 2022. The ratio between the prescription of amoxicillin and amoxicillin–clavulanic acid is only 0.48 in Italy, while the target is >119.

Although the Infectious Diseases Society of America (IDSA) has identified pediatrics as a priority area for further research into the effectiveness of stewardship activities, there is still a paucity of literature on the topic [21]. In recent years, the CDC has issued several documents, including one that helps physicians improve antimicrobial stewardship programs in both hospital and outpatient settings [7,22]. The COVID-19 pandemic has affected Italy since February 2020. The lower number of ED visits in July–September compared to the first months of 2020 (4610 vs. 2221) probably reflects both the physiological decline in pediatric infectious diseases in summer and the decrease in ED visits for common illnesses, mainly due to fear of COVID-19 infection [23]. Children who presented to the ED in the summer had more severe pathologies with a higher need for hospitalization and higher use of parenteral drugs and broad-spectrum antibiotics compared to winter (Table 1 and Table 2) [24]. These elements could not be predicted at the time of study design and may have influenced the results.

Despite these differences, the study team reviewed each individual case and assigned a final score, which allowed a precise analysis of the different clinical presentations.

Globally, 94% of infectious diseases in the first period were treated with a specific face-to-face meeting during the intervention phase, followed by specific written digital material. This led to an overall improvement in prescribing for all common pediatric infections (Figure 2).

The statistically significant difference observed in the main aspects of antibiotic prescribing (Table 3) shows how relevant the impact of a stewardship intervention can be in the pediatric ED setting. The dissemination of digital material was effective and useful and was also available at the patient’s bed.

The molecules prescribed in both periods reflect the national data: penicillins plus inhibitor (amoxicillin–clavulanic acid in primis) are the most commonly used drug combinations, followed by broad-spectrum penicillin (amoxicillin) and third-generation cephalosporins and macrolides, confirming the tendency to prescribe molecules with greater efficacy than necessary (Table 2 and Table 3).

However, the improvement observed in the control period (excess of drug of choice in 75% PRE vs. 54% POST cases) seems promising, especially considering that children treated in the control period had a more severe clinical presentation, with a greater need for hospitalization and parenteral antibiotic therapy compared to the first period. The amoxicillin/amoxicillin–clavulanic acid ratio of 0.6 in the first period was in line with national data (Northern Italy: 0.76; national average: 0.48). In the second period, the ratio was 0.4, reflecting the increased use of broad-spectrum antibiotics due to the more severe diseases being treated.

However, the focus on the treatment of some conditions has led to an improvement in prescribing accuracy. For example, the reversal of the amoxicillin/amoxicillin–clavulanic acid ratio observed in otitis media and pharyngotonsillitis and the use of high doses in otitis media were excellent and unexpected results (Figure 1).

The prescribed dose was lower than optimal in the first period and higher in the control period. This could be due to the higher winter incidence of diseases for which guidelines recommend high doses of antibiotics, such as ear infections and pneumonia, which are often treated with suboptimal doses. On the other hand, in the summer period, there is a higher incidence of diseases for which the standard dose is sufficient; instead, there is a tendency to use more generous doses of the drug (Table 3).

In pediatrics, however, it is particularly advisable to adhere to the guideline doses or to prescribe slightly higher doses, especially in the younger age groups, because of the difficulties often encountered in administering the drug if it is lost.

The duration of therapy in both periods (Table 3) may reflect the difficulties of the ED setting, including the need to obtain patient compliance. Further efforts are needed to get as close as possible to what is indicated in the guidelines, together with an action to educate the family about the usefulness of complying with the prescriptions: longer therapies, where indicated, aim at a greater eradication of pathogenic bacteria, reducing the risk of relapses and the emergence of resistances.

From the analysis of the data collected, we can draw observations that frame the main difficulties encountered by an ED physician when prescribing antibiotics, both in the PRE and POST periods, and highlight further areas for improvement.

In 13% of all prescriptions, the ED prescriber recommended antibiotic therapy only in the presence of a precise clinical evolution (e.g., persistence of fever after 24–72 h, worsening of symptoms). The inability to follow up the discharged patient and the questionable compliance of families to repeat medical examinations soon, even when explicitly indicated, could lead to unnecessary antibiotic administration.

Twenty-one percent of the total 1423 antibiotic prescriptions were given to children who presented to the ED with ongoing antibiotic therapy prescribed in the previous days by their family doctor: 72% of these prescriptions were confirmed unchanged. However, in 28% of the latter cases, there was no indication for antibiotic therapy.

These findings highlight a certain difficulty in changing previously initiated therapies, even when they are no longer considered necessary. This may be due to the difficulty of explaining the change to families and the fear of losing their trust, as well as the delicate relationship with the family pediatrician.

This is an area that needs further attention: in the fight against antibiotic resistance, we should stop any unnecessary therapy as soon as possible. From this point of view, greater interaction between emergency physicians and general practitioners is desirable. A strong point of the study was how all the doctors involved (pediatricians, ID specialists, trainees) participated in the work and meetings voluntarily and with interest and willingness to confront and improve. The meetings in small groups and the discussion of clinical cases were enriching moments of mutual learning between the different characters, which strengthened the relationships between the doctors and improved the diagnostic and therapeutic pathways in our hospital.

It also seems useful to highlight how an improvement in the IT resources available in our hospital could facilitate further and effective stewardship programs. The current IT program does not allow easy access to drug prescriptions, especially antibiotics [25]. The study team manually identified each case in the study among all instances of attending the ED. Easier and faster access to this information could certainly make it easier to analyze the appropriateness of antibiotic prescribing.

Study limitations

- The two study groups differed in age, site of infection, and severity of clinical presentation, reflecting the different winter/summer distribution of infectious diseases and the effects of the COVID-19 pandemic. This may have influenced the results.
- We did not report the number of patients seen again in the emergency department in the first 7 days and 30 days after the first visit.
- Our study did not evaluate the use of antibiotics in the total number of febrile children with access to the ED.
- We did not follow up the hospitalized children in their therapeutic process; therefore, we do not know if the ED therapeutic suggestion was continued or changed during hospitalization and what the patient’s outcome was.

## 5. Conclusions

The audit strategy in this study, based on interactions between an infectious disease team and prescribers, has proven to be a successful choice.

The educational stewardship intervention, albeit brief, used in this study, with educational meetings by pathology and dissemination of training materials that could be used at the patient’s bedside, resulted in improved prescriptive appropriateness already within a few months. Particularly with regard to very common conditions such as otitis media and pharyngotonsillitis, which represented the main targets of antibiotic prescribing in pediatric emergency rooms.

Further studies are needed to assess whether the improvement in prescribing is sustained over time and in the case of typical winter infectious diseases. In conclusion, an antimicrobial stewardship project is feasible and effective even in a specific and unique reality, such as a pediatric emergency department.

## Figures and Tables

**Table 1:** Study population: mean (SD) or frequencies (%); *p* value; type of test.

	PRE-Intervention (Jan–Mar)	POST-Intervention (Jul–Sep)	*p*	TEST
N of patients accessing the ED	4610	2221		
N of ATBs prescriptions (%)	1128 (24.4)	293 (13.2)		
Median age, years (SD)	4.38 (3.50)	5.43 (4.36)	<0.001	*t*-test
Route of administration (%)			0.002	exact
EV prescription	125 (11.1)	54 (18.4)		
OS prescription	1002 (88.8)	239 (81.6)		
Destination (%)			0.009	exact
Discharge to home	978 (86.7)	242 (82.6)		
Hospital recovery	148 (13.1)	51 (17.4)		
Site of infection (%)				
Abdomen	21 (1.9)	13 (4.4)		
Upper airways	610 (54.1)	114 (38.9)		
Lower airways	275 (24.4)	19 (6.5)		
Bone	6 (0.5)	2 (0.7)		
Urinary tract	84 (7.4)	55 (18.8)		
Skin and soft tissues	61 (5.4)	59 (20.1)		
CNS	7 (0.6)	0 (0.0)		
Lymphadenitis	24 (2.1)	11 (3.8)		
Other	40 (3.5)	20 (6.8)		

Abbreviations: ATBs, antibiotics; CNS, central nervous system; EV, endovenous; Jan, January; Jul, July; Mar, March; N, number; Sep, September.

**Table 2:** Comparison of the prescribed group of molecules and spectra in the pre-post study.

Prescribed Antibiotics-All Diseases, PRE-POST Intervention			
	PRE (%)		POST (%)
Amoxicillin	279 (24.9)		64 (22.1)
Amoxicillin–clavulanic acid	431 (38.5)		147 (50.7)
Cephalosporins	204 (18.2)		54 (18.6)
Macrolides	180 (16.1)		12 (4.1)
Other penicillins	10 (0.9)		2 (0.7)
Other	16 (1.4)		11 (3.8)
Ratio of amoxicillin/amoxicillin–clavulanic acid	0.64		0.43
**Use of Antibiotics for Spectrum Groups**			
**SPECTRUM GROUPS**	**PRE (%)**	**POST (%)**	***p***
BROAD-SPECTRUM (Penicillins + β-lactamase inhibitors, III and IV generation cephalosporins, Carbapenems, Fluoroquinolones, Fosfomycin)	652 (58.1)	207 (71.1)	<0.001
NARROW-SPECTRUM (Aminopenicillins, I and II generation cephalosporins, Glycopeptides, Macrolides, Aminoglycosides, Metronidazole, Trimethoprim/sulfamethoxazole)	471 (41.9)	84 (28.9)	

**Table 3:** Item score assignment by grade of agreement in the pre-post evaluation.

Item of Evaluation	Grade of Agreement	PRE n (%)	POST n (%)	*p*
Overall prescriptive adequacy	A	135 (13.3)	101 (43.5)	<0.001
	B	543 (53.6)	96 (41.4)	
	C	336 (33.1)	35 (15.1)	
Need for antibiotics	A	550 (53.4)	158 (68.1)	<0.001
	B	211 (20.5)	41 (17.7)	
	C	268 (26.0)	33 (14.2)	
Adequacy of the spectrum	A	485 (55.1)	141 (63.5)	<0.001
	B	272 (30.9)	78 (35.1)	
	C	123 (14.0)	3 (1.4)	
Adequacy of the molecule	A	473 (53.8)	137 (61.7)	<0.001
	B	283 (32.2)	78 (35.1)	
	C	123 (14.0)	7 (3.2)	
Adequacy of the dose	A	585 (74.8)	152 (72.4)	0.454
	B	147 (18.8)	47 (22.4)	
	C	50 (6.4)	11 (5.2)	
Adequacy of the duration	A	574 (81.2)	143 (83.1)	0.824
	B	130 (18.4)	29 (16.9)	
	C	3 (0.4)	0 (0.0)	
**Evaluation of Excess/Defect for Items of Grade “B” or “C”**				
**Item of Evaluation**	**Defect/Excess**	**PRE n (%)**	**POST n (%)**	***p***
Adequacy of the molecule (%)	Defect	100 (24.8)	29 (35.8)	0.041
	Excess	303 (75.2)	52 (64.2)	
Adequacy of the dose (%)	Defect	139 (69.8)	30 (35.7)	<0.001
	Excess	60 (30.2)	54 (64.3)	
Adequacy of the duration (%)	Defect	101 (76.5)	33 (56.9)	0.006
	Excess	31 (23.5)	25 (43.1)	

## Data Availability

The data presented in this study are available on request from the corresponding author due to the ongoing study.

## Supplementary Materials

The following supporting information can be downloaded at: https://www.mdpi.com/article/10.3390/children12010046/s1.
