# Supplementary material for: Antimicrobial Stewardship in the Pediatric Emergency Department: An Observational Pre-Post Study
Source: Children (Basel). 2024 Dec 30;12(1):46. doi: 10.3390/children12010046 (PMC11764019; doi:10.3390/children12010046)
Supplement: Supplementary file 1 [file children-12-00046-s001.zip › children-3377429-supplementary.pdf]

# EVALUATION CRITERIA

Each antibiotic prescription was evaluated retrospectively by the stewardship team. Specifically, the aspects of prescribing evaluated were as follows:

- Overall appropriateness of prescribing
- The need for antibiotic therapy according to the diagnosis hypothesized by the emergency room physician.
- The appropriateness of the spectrum of the molecule chosen
- The appropriateness of the molecule, concerning the site of infection and the patient's age
- The appropriateness of the prescribed dose
- The appropriateness of the duration indicated for the pathology

The appropriateness assessment used a score defined as follows:

A. complete agreement between the prescribing physician and the stewardship team
B. partial agreement between the prescribing physician and the stewardship team (appropriateness not considered complete)
C. disagreement between the prescribing physician and the stewardship team
